# External Quality Assessment for the Detection of *Chlamydia trachomatis* in Urine Using Molecular Techniques in Belgium

**DOI:** 10.1155/2015/835261

**Published:** 2015-06-25

**Authors:** Bernard China, Kris Vernelen

**Affiliations:** Quality of Medical Laboratories, Scientific Institute of Public Health, J. Wytsman 14, 1050 Brussels, Belgium

## Abstract

*Chlamydia trachomatis* is a major cause of sexually transmitted bacterial disease worldwide. *C. trachomatis* is an intracellular bacterium and its growth *in vitro* requires cell culture facilities. The diagnosis is based on antigen detection and more recently on molecular nucleic acid amplification techniques (NAAT) that are considered fast, sensitive, and specific. In Belgium, External Quality Assessment (EQA) for the detection of *C. trachomatis* in urine by NAAT was introduced in 2008. From January 2008 to June 2012, nine surveys were organized. Fifty-eight laboratories participated in at least one survey. The EQA panels included positive and negative samples. The overall accuracy was 75.4%, the overall specificity was 97.6%, and the overall sensitivity was 71.4%. Two major issues were observed: the low sensitivity (45.3%) for the detection of low concentration samples and the incapacity of several methods to detect the Swedish variant of *C. trachomatis*. The reassuring point was that the overall proficiency of the Belgian laboratories tended to improve over time.

## 1. Introduction

*Chlamydia trachomatis* infection is the most prevalent sexually transmitted bacterial disease and is, therefore, a significant global health problem. It is estimated that 90 million cases occur annually worldwide [1]. The number of those infected is likely to be much higher because most of the infected people were asymptomatic [2]. In Belgium, the number of diagnosed cases was 3314 in 2010 with an incidence rate of 30.8/100 000 people [3].

*C. trachomatis* is a nonmotile obligate intracellular bacterium characterized by a unique biphasic developmental cycle [4]. Based on the antigenic reactivity of the OMP (Outer Membrane Proteins),* C. trachomatis* is currently divided into 18 serotypes. Serotypes A, B, Ba, and C are generally associated with blinding trachoma and serotypes D to K are responsible for causing nondisseminating sexually transmitted infections. These 12 serotypes (A, B, Ba, C and D–K) are all naturally restricted to infection of genital or ocular epithelial cells and have not been observed as invasive [5]. By contrast serotypes L1, L2, L2a, and L3 cause a number of invasive and systemic sexually transmitted infections normally found in the tropics, known as lymphogranuloma venereum (LGV) [6].

The type and anatomical site of specimen collection for laboratory diagnosis of* C. trachomatis* infection depend on both the clinical picture and the laboratory test selection [4]. Noninvasively collected specimens such as first-void urine and vulvogenital swab specimen are excellent for the diagnosis of* C. trachomatis* genital tract infection by nucleic acid amplification techniques (NAAT). Due to their high sensitivity and specificity, NAAT are the tests of choice for diagnosis of genital* C. trachomatis* infections in routine clinical laboratories. NAAT can be used to detect* C. trachomatis* without a pelvic examination or intrauteral swab specimen by testing self- or clinician-collected vaginal swab or urine [4]. In many evaluations, NAAT detected 20 to 30% more positive specimens than could be detected by non-NAAT technologies. Licensed NAAT for detection of* C. trachomatis* include (i) PCR-based methods either conventional PCR methods such as Roche Amplicor or the real time PCR methods such as Roche TaqMan (Roche Diagnostics, Basel, Switzerland) and the Abbott real time (TM) CT or CT/NG assay (Abbott, Abbott Park, IL, USA), (ii) Transcription Mediated Amplification (TMA) such as APTIMA (Gen-Probe Inc., San Diego, CA), and (iii) the strand displacement amplification (SDA) such as the BD ProbeTec method (Becton Dickinson and Company, Diagnostic Systems, Franklin Lakes, NJ).

In 2006, a new variant of* C. trachomatis* was described in Sweden presenting a 377 bp deletion of the plasmid DNA [7]. Since some NAAT are based on the detection plasmid specific DNA regions, some false negative results can occur [7].

In Belgium, the reimbursement by the social security insurance of the detection of microbes using molecular techniques by medical laboratories was specifically introduced into the legislation in 2008 [8]. The reimbursement was coupled to the obtaining of ISO15189 [9] accreditation and to the participation in External Quality Assessment (EQA). Since 2008, the Belgian Scientific Institute of Public Health (IPH) has organized the EQA for these laboratories including the detection of* C. trachomatis* in urine and swabs. The present paper describes the results obtained from 2008 to 2012 for the detection of* C. trachomatis* in urine using NAAT.

## 2. Material and Methods

### 2.1. The Samples

From 2008 to 2012, 9 EQA sample panels were provided to the participants. It means two panels per year (CTA and CTB) except for 2012 where only one panel was provided. These panels consisted of urine and simulated swabs samples. In this paper only urine samples were considered. The EQA samples (Table 1) were provided by Quality Control for Molecular Diagnostics (QCMD, Glasgow, Scotland). QCMD is accredited under the international standard ISO17043 [10] for the provision of EQA. The* C. trachomatis* strains used were either* C. trachomatis* LGV serovar L2 or* C. trachomatis* Swedish variant [7]. Samples were lyophilized and required reconstitution following the instructions manual provided with the samples. Laboratories were instructed to process the samples as routine urine or swab samples.

The samples varied in their amount of target* C. trachomatis* DNA. Samples could be negative meaning that no* C. trachomatis* DNA molecule was present.

### 2.2. The Participants

In Belgium, EQA is mandatory for the clinical biology laboratories [12]. In 2008, some parameters of molecular microbiology were introduced into the scope of the EQA scheme [13]. From 2008 to 2012, fifty-eight Belgian laboratories were registered yearly to the EQA for the detection of* C. trachomatis* using molecular techniques.

### 2.3. The Procedure

The registered laboratories received the EQA samples (Table 1) and were given around one month time to return their results to QCMD via the QCMD web page (http://www.qcmd.org/). Each participant possessed its own login and password to have access to their personal participant area. Information about the extraction method, the detection method, the testing results, and any encountered problems was entered online.

After the closure of the results return period, participants received an individual and an overall final report. At the end of a cycle, the Belgian participants received an annual report including their results for all the panels of the previous year.

### 2.4. Evaluation

Sample status is assigned by peer-group consensus based on the qualitative results returned by all participants in the full EQA program. It was not a measure of the “strength” of a positive sample nor was it technology-dependent and was used solely for the scoring of the EQA data. The rationale for the sample status was as follows.

 Frequently detected: more than 95% of datasets recorded the correct positive result.
 Detected: between 65 and 95% of the datasets recorded the correct positive result.
 Infrequently detected: less than 65% of the datasets recorded the correct positive result.
 Negative: a sample that does not contain the target produced an unequivocal negative result.

A scoring system was established by QCMD for individual performance assessment (Table 2). For each correct answer the lab received a score of 0. A false positive result was scored as +3. A false negative result was scored as +1 for infrequently detected samples; +2 for detected samples; and +3 for frequently detected samples. Therefore, using this scoring system, the lowest score is the best. A “not determined” result was not scored. For IPH, a false result was considered a clinically relevant fault in two cases: a false positive result and a false negative result for a frequently detected sample. The participant encoding a clinically relevant fault is susceptible to receive an official claim from the IPH. This claim must be treated as a nonconformity in their quality management system.

### 2.5. The Methods

The different methods used by the Belgian laboratories during the surveys were listed in Table 3.

## 3. Results

### 3.1. Participants

Between January 2008 and June 2012, nine surveys were organized.

The number of participating laboratories ranged from 51 to 54 per survey. The percentage of responding laboratories ranged from 85 to 96%. The number of datasets returned to QCMD was always higher than the number of responding laboratories indicating that some laboratories introduced more than one dataset. Indeed, EQA is very often an occasion for the laboratories to validate new methods.

From 2008 to 2012, 58 different laboratories responded; among them 33 (56.9%) participated in 9 surveys, 5 (8.6%) in 8 surveys, 6 (10.3%) in 7 surveys, 1 (1.7%) in 6 surveys, 4 (6.9%) in 5 surveys, 2 (3.4%) in 4 surveys, 1 (1.7%) in 3 surveys, 3 (5.2%) in 2 surveys, and 3 (5.2%) in 1 survey.

### 3.2. Proficiency for Urine Samples

**Figure 1:**
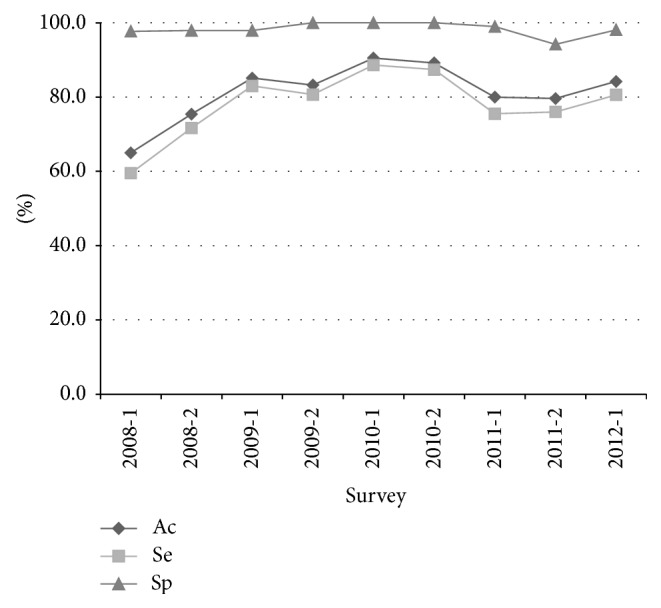
Parameters per survey. Ac: accuracy; Se: sensitivity; Sp: specificity.

The overall number of Belgian results was 2917. The returned results were always qualitative (presence or absence) results. The number of correct results (accuracy) was 2198/2917 (75.4%). The number of incorrect results was 719/2917 (24.6%). Of the incorrect results, the number of false positive results was 6/719 (0.8%) and the number of false negative results was 693/719 (96.4%). The number of inhibition results was 20/719 (2.8%). The number of negative and frequently detected samples was 445 and 747, respectively. The number of clinically relevant faults was 61/1192 (5.1%) including 6/445 (1.3%) false positive and 55/747 (7.4%) false negative. The overall sensitivity and specificity were 71.4% (1764/2472) and 97.6% (434/445), respectively. When the yearly evolution was considered (Figure 1), a general increase both in sensitivity and in specificity was observed from 2008 to 2012. For the 2011-1 survey, a decrease in sensitivity was observed partially due to the presence of a sample with a very low copy number (CTADNA11-01).

**Figure 2:**
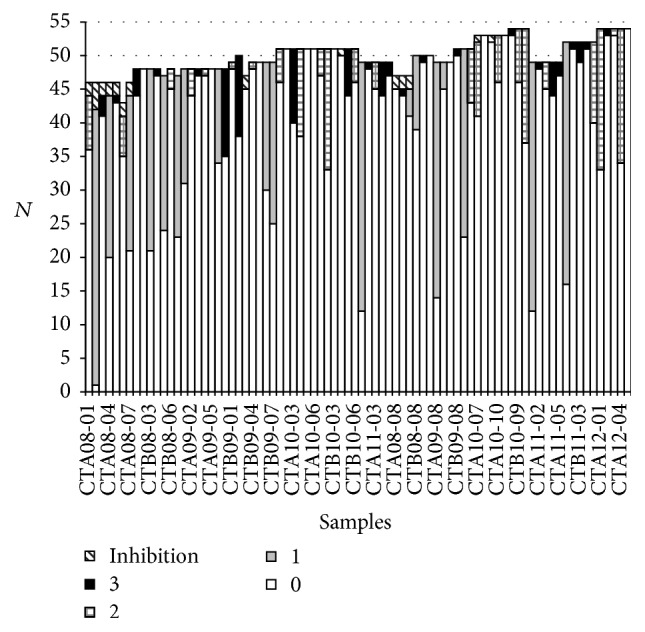
Score of the laboratories per sample. The number (*N*) of encoded results per sample was shown with the indication of the attributed score. 0 for a good answer, 1 for a wrong answer for an infrequently detected sample, 2 for a wrong answer for a detected sample, and 3 for a wrong answer for a frequently detected or a negative sample. The number of inhibition results was also indicated.

The proficiency per sample is shown in Figure 2. The percentage of correct answers ranged widely from 2.1% for sample CTA08-02 to 100% for samples CTA10-02, CTA10-05, CTA10-06, CTB10-03, and CTA12-05.

The samples with regard to the amount of target genome present were divided into frequently detected, detected, infrequently detected, and negative sample status. The percentage of correct answers was 92.4% for frequently detected, 86.4% for detected, 45.3% for infrequently detected, and 97.5% for negative samples, respectively.

### 3.3. Comparison of the Detection Methods

When the detection methods used were considered (Table 4), the most frequently used methods were Roche Cobas TaqMan CT method (23.5%), the BD ProbeTec ET system (17.7%), and the Roche Cobas Amplicor CT/NG method (13.4%). The methods used in less than 5 series of results were not considered. The methods can be ranked regarding their accuracy results (the % of correct answers). The accuracy ranged from 64.1 (Roche Amplicor CT/NG) to 93.7% (Nanogen Chlamydia tr. Q-PCR Alert).

### 3.4. The Swedish Variant

During the nine surveys from 2008 to 2012, 8 samples (1 infrequently detected, 1 detected, and six frequently detected) consisted of the Swedish variant of* C. trachomatis*. The results indicated that only 307 out of 401 (76.5%) answers were correct. The study of the methods used to analyze these samples (Table 3) showed that some kits (Roche Amplicor and Roche Cobas Amplicor) were unable to detect this* C. trachomatis* Swedish variant.

### 3.5. The Scores of the Laboratories

**Figure 3:**
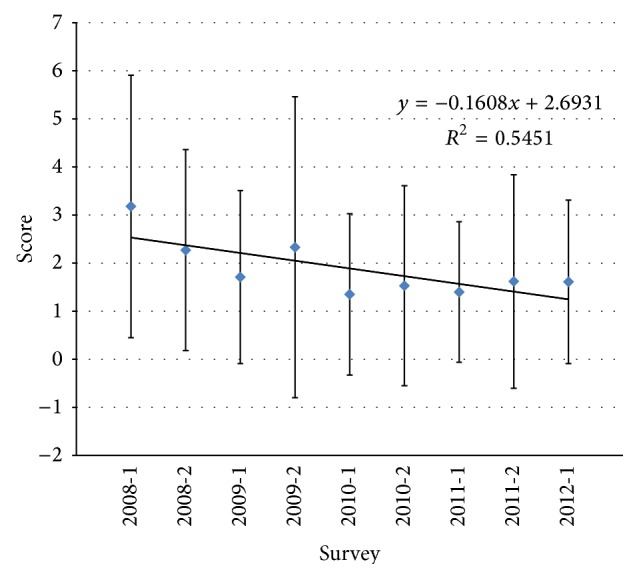
Means and standard deviation of the scores of the laboratories per survey for the detection of* C. trachomatis* using molecular techniques. The curves indicated the trends over the time.

The laboratories were evaluated by the attribution of a score (Table 2). When the IPH scores of the laboratories were considered over the time (Figure 3), the trend was a decrease in the scores indicating an increase in the proficiency of the Belgian laboratories.

## 4. Discussion

*Chlamydia trachomatis* is responsible for sexually transmitted disease. In Belgium, the number of diagnosed cases increased from 691 in 1997 to 3314 in 2010 [3].* C. trachomatis* is a fastidious bacterium and its growth can be problematic. Classically the diagnosis of* C. trachomatis* was based on the detection of specific antigens. Therefore, the introduction of molecular techniques for the detection of* C. trachomatis* in urine or swabs was a helpful progress in the diagnosis of* C. trachomatis* infection. Indeed, clinical evaluation of the NAAT has shown that they are more sensitive than culture and other methods including microscopy, antigen detection, and nucleic acid hybridization [4].

In Belgium, mandatory EQA for the detection of* C. trachomatis* by NAAT was introduced in 2008 [8]. The laboratories must participate in the External Quality Assessment (EQA) organized by the Scientific Institute of Public Health. In this context, since 2008, the IPH has organized the EQA for the detection of* C. trachomatis* in urine and swabs in collaboration with QCMD.

Between January 2008 and December 2012, nine surveys were organized. Only urine results are discussed in this paper since swabs results gave no additional information. For Belgium, 58 laboratories participated in at least one survey. These laboratories are clinical biology laboratories according to the Belgian legislation [12], representing about 30% of the total of the Belgian clinical biology laboratories. Among the IPH proposed EQA microbiological parameters for the detection by molecular techniques,* C. trachomatis* is the parameter for which the biggest number of laboratories was registered.

The Belgian participants represented around 28% of the total number of the participants in QCMD survey.

For* C. trachomatis* the encoded results were qualitative results although some NAAT methods allow quantification.

When the results were considered in relation to the type of samples, two major issues were observed. First, the sensitivity was low (below 50%) for the detection of infrequently detected samples. The results indicated that when the number of copies per vial was under 60, the detection power was low. It raises the question of the clinical cut-off. It seems clear that in case of a true infection the level of* C. trachomatis* is expected to be high.

Second, the sensitivity for the detection of the Swedish variant strain of* C. trachomatis* is also low (around 75%). It is particularly clear (Table 3) that some methods were unable to detect this variant. This is due to the fact that the Swedish variant has a 377 bp deletion in the cryptic plasmid [11]. Therefore detection kits such as the Roche (Cobas) Amplicor CT/NG kit that target the deleted region were unable to detect the Swedish variant [14, 15]. In our study, the Roche Cobas/Amplicor CT/NG kits were also unable to detect the Swedish variant even when present in large amounts. It should be noted that the insert of these kits contains the mention: “The Amplicor CT/NG Test or the Cobas Amplicor CT/NG test for* Chlamydia trachomatis* will not detect plasmid-free variants of* C. trachomatis*.” For the Roche Cobas TaqMan CT/NG kit, it is interesting to notice that, in 2008 and in the first survey of 2009, the kit was unable to detect the variant DNA but due to an improvement of the kit (v2.0), the further samples were well detected. Therefore, it is important that all the laboratories do not use the same method to detect* C. trachomatis* to minimize the fact that the presence of a new variant will be not detected. Moreover, the case of the Swedish variant underlines the fact that the EQA is a powerful means to detect the failing of kits or methods on certain samples.

The new Swedish variant (nvCT) represents 20 to 64% of the detected Chlamydia cases in Sweden [14]. Although the nvCT has been detected in Norway, Finland, and Denmark [16], only a few cases of nvCT have been reported outside the Nordic countries [17–19].

Among the most used methods, the Abbott real time PCR CT/NG and the Roche Cobas TaqMan CT/NG v2.0 gave the best results with an accuracy of 87.4 and 90.1%, respectively. It is not clear from the results that the homemade methods were less proficient than the commercial kits.

When the technologies used were compared, real time PCR methods gave better results than SDA methods or than conventional PCR methods. The TMA (Transcription Mediated Amplification) methodology was only used by one participant in two surveys and was not considered here.

Finally, when the proficiency over time was considered, a decrease in the penalty points scored was observed indicating an increase in proficiency. This is encouraging for the future and that fully justifies the need of EQA.

EQA allows comparing the proficiency of the laboratories but also of the methodologies. For a small country such as Belgium, it is also useful to participate in international surveys including other countries in order to be able to compare the result of a lab with other laboratories using the same methodology. It is particularly true for the methods that are not frequently used.

The major issue for the EQA is to have samples as close as possible to clinical samples. But these samples must also be homogeneous and stable. Moreover, the pre- and postanalytical process should also be assessed. For the preanalytical phase, the sampling and the transport conditions are important. The postanalytical evaluation is particularly important in case of low contaminated samples. It is not evident that all the laboratories will give the same answer to the clinician. The determination of an analytical and a clinical cut-off is sometimes required. Nevertheless, the improvement of the quality of the diagnosis is our priority and a powerful EQA is a major tool in this goal.

## 5. Conclusion

A correct diagnosis is closely related to powerful diagnostic tools. The proficiency of these methods can be evaluated using External Quality Assessment. In Belgium, for many years, the participation in EQA is mandatory for the medical laboratories. Moreover, for molecular testing, the ISO15189 accreditation is also required. The results of the EQA for the detection of* C. trachomatis* in urine using molecular methods revealed a low sensitivity (71.4%) but a good specificity (97.6%). The low sensitivity is mainly related to the lack of detection of the Swedish variant by several methods. Nevertheless, the situation improved with time indicating that the laboratories and the companies make effort to guaranty the best result.

## Figures and Tables

**Table 1:** *C. trachomatis* EQA urine samples.

Name	Year	Content (copies/vial)	Status	Penalty	Name	Year	Content (copies/vial)	Status	Penalty
CTA08-01	*2008 *	570	Detected	2	CTA10-01	*2010 *	280	Detected	2
CTA08-02	*2008 *	6	Infrequently detected	1	CTA10-02	*2010 *	5700	Frequently detected	3
CTA08-03	*2008 *	5700	Frequently detected	3	CTA10-03^b^	*2010 *	ND	Frequently detected	3
CTA08-04^a^	*2008 *	57	Infrequently detected	1	CTA10-04	*2010 *	57	Infrequently detected	1
CTA08-05	*2008 *	0	Negative	3	CTA10-05	*2010 *	280	Detected	2
CTA08-06	*2008 *	570	Detected	2	CTA10-06	*2010 *	0	Negative	3
CTA08-07	*2008 *	57	Infrequently detected	1	CTB10-01	*2010 *	280	Detected	2
CTB08-01	*2008 *	570	Detected	2	CTB10-02	*2010 *	57	Detected	2
CTB08-02	*2008 *	5700	Frequently detected	3	CTB10-03	*2010 *	5700	Frequently detected	3
CTB08-03	*2008 *	57	Infrequently detected	1	CTB10-04	*2010 *	0	Negative	3
CTB08-04	*2008 *	0	Negative	3	CTB10-05^b^	*2010 *	ND	Frequently detected	3
CTB08-05	*2008 *	57	Infrequently detected	1	CTB10-06	*2010 *	280	Detected	2
CTB08-06	*2008 *	570	Detected	2	CTA11-01	*2011 *	50	Infrequently detected	1
CTB08-07^b^	*2008 *	ND	Infrequently detected	1	CTA11-02	*2011 *	0	Negative	3
CTA09-01	*2009 *	57	Infrequently detected	1	CTA11-03	*2011 *	400	Detected	2
CTA09-02	*2009 *	570	Detected	2	CTA11-04^b^	*2011 *	10^6^	Frequently detected	3
CTA09-03	*2009 *	0	Negative	3	CTA11-05	*2011 *	5000	Frequently detected	3
CTA09-04	*2009 *	570	Detected	2	CTB11-01	2011	50	Infrequently detected	1
CTA09-05	*2009 *	5700	Frequently detected	3	CTB11-02	2011	5000	Frequently detected	3
CTA09-06	*2009 *	57	Infrequently detected	1	CTB11-03^b^	2011	10^6^	Frequently detected	3
CTA09-07^b^	*2009 *	ND	Frequently detected	3	CTB11-04	2011	0	Negative	3
CTB09-01	*2009 *	570	Detected	2	CTB11-05	2011	400	Detected	2
CTB09-02^b^	*2009 *	ND	Frequently detected	3	CTA12-01^b^	2012	2000	Detected	2
CTB09-03	*2009 *	0	Negative	3	CTA12-02	2012	0	Negative	3
CTB09-04	*2009 *	570	Detected	2	CTA12-03	2012	1000	Detected	2
CTB09-05	*2009 *	5700	Frequently detected	3	CTA12-04	2012	250	Detected	2
CTB09-06^a^	*2009 *	57	Infrequently detected	1	CTA12-05	2012	4000	Frequently detected	3
CTB09-07	*2009 *	57	Infrequently detected	1					

^a^ *C. trachomatis* + *N. gonorrhoeae* (from 6 · 10^5^ to 5 · 10^6^ CFU/vial).

^b^Swedish variant missing 377 bp of the cryptic plasmid [11].

**Table 2:** Scoring system.

Sample status	Negative	Positive	Not determined
Frequently detected	+3	0	Not scored
Detected	+2	0	Not scored
Infrequently detected	+1	0	Not scored
Negative	0	+3	Not scored

**Table 3:** Method used per year and per participant.

Methods	2008a	2008b	2009	2010	2011a	2011b	2012
Real time PCR							
Abbott real time CT^1^	1	2	2	2	2	2	2
Abbott real time CT/NG^1^		1	2	8	10	13	15
Nanogen Chlamydia tr. Q-PCR Alert kit^2^		1	1	1	1	1	1
Shangai bio-tech IMtec CT real time PCR kit^3^		1					
Roche Cobas TaqMan CT^4^	4	4	9				
Roche Cobas TaqMan CT v2.0 Roche^4^				14	14	12	12
Roche Cobas TaqMan CT/NG^4^			1			3	7
Qiagen artus *C. trachomatis* PCR kit^5^	5	5	7	5	5	5	4
Diagenode DIA-CT-050^6^				1	1	2	1
Real time in house	1	3		5	3	4	4
Gen-Probe Aptima combo 2 Assay^7^		1					
Gen-Probe PACE CT^7^		1					
Gen-Probe PACE 2 CT^7^							
SDA							
Becton Dickinson ProbeTec ET^8^	11	10	9	7	6	6	5
PCR-ELISA							
Roche Amplicor CT^4^	1						
Roche Amplicor CT/NG^4^	12	11		8	4	2	3
Roche Cobas Amplicor CT/NG^4^	10	10	17	3	4	1	1
Other							
Nasba in house	2						
Hain Lifescience Genoquick CT^9^			1				
Total of the participants	47	50	48	54	50	51	55

^1^Abbott laboratories, Abbott Park, IL, USA.

^2^Nanogen Advanced Diagnostics, Trezzano sul Naviglio, Italy.

^3^IMTEC, Berlin, Germany.

^4^Roche Molecular diagnostics, Pleasanton, USA.

^5^Qiagen, Venlo, Netherlands.

^6^Diagenode, Liège, Belgium.

^7^Gen-Probe Incorporated, San Diego, USA.

^8^Becton Dickinson and Co, Sparks, USA.

^9^Hain Lifesceince GmBH, Nehren, Germany.

**Table 4:** The proficiency per detection methods.

#	Method	*N* ^1^	Spls^2^	+^3^	−^4^	Ac^5^ (%)	Se^6^ (%)	Sp^7^ (%)	Swedish variant									
									B08-07	A09-07	B09-02	A10-03	B10-05	A11-04	B11-03	A12-01	Total	%
1	Nanogen Chlamydia tr. Q-PCR Alert	8	48	40	8	93.70	90.00	100.00	1/1	1/1	1/1	1/1	1/1	1/1	1/1	1/1	8/8	100.0
2	Roche Cobas TaqMan CT^8^	93	547	454	93	90.10	88.10	100.00	0/4	0/9	10/10	14/14	14/14	14/14	12/12	6/12	76/89	85.4
3	Abbott real time CT/NG	56	303	247	56	87.40	92.30	98.20	1/1	2/2	2/2	5/5	8/8	10/10	13/13	11/15	52/56	92.9
4	Abbott real time CT	17	103	86	17	83.50	80.20	100.00	2/2	1/2	2/2	2/2	2/2	2/2	2/2	1/2	14/16	87.5
5	Qiagen artus *C. trachomatis* Plus	48	297	249	48	81.80	78.30	100.00	5/5	7/7	6/6	6/6	5/5	5/5	5/5	3/4	42/43	97.7
6	BD Diagnostics BD ProbeTec ET	70	441	371	70	75.90	78.20	95.70	10/10	9/9	8/8	8/8	7/7	6/6	6/6	2/5	56/59	94.9
7	Roche Cobas Amplicor CT/NG	53	359	306	53	75.20	71.20	98.10	0/10	NA	4/8	0/4	0/2	0/2	0/1	NA	4/27	14.8
8	Roche Amplicor CT/NG	51	329	278	51	64.10	59.40	94.10	0/11	4/17	1/8	0/7	0/5	0/3	0/2	0/3	5/56	8.9
	Total	396	2427	2031	396	80.2	78.8	98.0	22/47	24/47	36/48	39/50	42/49	41/46	39/41	26/42	257/354	72.6
									46.8%	51.1%	75.0%	78.0%	85.7%	89.1%	95.1%	61.9%	72.6%	

^1^ *N*: The number of samples panels analyzed using this method.

^2^The number of analyzed samples.

^3^+: the number of positive samples analyzed.

^4^−: the number of negative samples analyzed.

^5^Ac: accuracy = percentage of correct results.

^6^Se: sensitivity percentage of correct results for positive samples.

^7^Sp: specificity = percentage of correct results for negative samples.

^8^Roche Cobas TaqMan CT (for 2008 and first survey of 2009) and Roche Cobas TaqMan CT v2.0 (since second survey of 2009).

NA: not applicable.
